# Epileptogenicity Maps of Intracerebral Fast Activities (60–100 Hz) at Seizure Onset in Epilepsy Surgery Candidates

**DOI:** 10.3389/fneur.2019.01263

**Published:** 2019-11-28

**Authors:** Anne-Sophie Job, Olivier David, Lorella Minotti, Fabrice Bartolomei, Stephan Chabardès, Philippe Kahane

**Affiliations:** ^1^Inserm, U1216, CHU Grenoble Alpes, Grenoble Institut Neurosciences, Université Grenoble Alpes, Grenoble, France; ^2^INS, Inserm, U1106, Marseille, France; ^3^Neurophysiology Departement, La Timone Hospital, Marseille, France

**Keywords:** epilepsy surgery, SEEG, epileptogenic zone, HFO, refractory epilepsy

## Abstract

Fast activities (FA) at seizure onset have been increasingly described as a useful signature of the epileptogenic zone (EZ) in patients undergoing intracranial EEG recordings. Different computer-based signal analysis methods have thus been developed for objectively quantifying ictal FA. Whether these methods detect FA in all forms of focal epilepsies, whether they provide similar information than visual analysis (VA), and whether they might help for the surgical decision remain crucial issues. We thus conducted a retrospective study in 21 consecutive patients suffering from drug-resistant seizures studied by SEEG recordings. Ictal FA were quantified using the Epileptogenicity Maps (EM) method that we recently developed and which generates, by adopting a neuroimaging approach, statistical parametric maps of FA ranging from 60 to 100 Hz (FA^60−100^). Ictal FA were analyzed blindly using VA and EM, and the prognostic significance of removing areas exhibiting FA^60−100^ at seizure onset was evaluated. A significant ictal FA^60−100^ activation was found in all patients, and in 92.6% of all the 68 seizures recorded, whatever the epilepsy type. The overlap ratio (OR) between VA and EM was significantly better for defining the regions spared at seizure onset than those from which seizure arose (*p* < 0.001), especially in temporal or temporal “plus” epilepsies. EM and VA were much more discordant to define the EZ, with a mean number of electrode contacts involved at seizure onset significantly higher with EM than with VA (*p* = <0.0001). Seizure outcome correlated with the resection ratio for FA^60−100^, which was significantly higher in seizure-free (Engel's class Ia) than in non seizure-free patients (class Ic-IV) (*p* = 0.048). The quantification of FA at seizure onset can bring information additional to clinical expertise that might contribute to define accurately the cortical region to be resected.

## Introduction

The primary aim of epilepsy surgery is to remove the epileptogenic zone (EZ), i.e., the minimum amount of cortex that must be resected to produce seizure freedom. The identification of the EZ is a difficult process, which requires intracranial EEG (iEEG) recordings in 25–50% of the cases. However, even when using such iEEG information, epilepsy surgery still fails in a substantial ratio of patients (1). This means that iEEG criteria used for identifying the epileptogenic brain tissue are not clearly determined nor understood.

Currently, iEEG demonstration of the seizure-onset zone—the cortical areas from where seizures start—offers a well-accepted approximation of the EZ. Traditionally, the identification of the EZ is done visually, and particular attention is paid on the classical low-voltage fast activity, which is the most characteristic iEEG seizure-onset pattern across all forms of focal epilepsies (2–4). By using signal analysis techniques, this pattern has been shown to be made of fast activities (FA) ranging from 20 to 200 Hz (5–13). Recent improvements in the acquisition technology have even shown that such activities can be as fast as 400 Hz (14) and even more (15). Importantly, the resection of brain regions exhibiting FA at seizure onset seems to predict a favorable surgical outcome (5, 11, 15–18). This paves the way to the development of quantitative FA-based indices to guide epilepsy surgery.

In this context, an innovative method was proposed by the group of Marseille, where spectral and temporal information of stereotactic intracerebral EEG (SEEG) signals were mixed together to provide an index—named *Epileptogenicity Index* (EI)—quantifying the implication of each cortical site in seizure onset and early propagation (19). Keeping the same basic principles of EI determination during SEEG recordings, we proposed another quantification of epileptogenicity by adopting a neuroimaging approach in order to generate statistical parametric maps of FA, named *Epileptogenicity Maps* (EM) (7). The method is based on spectral analysis of FA ranging from 60 to 100 Hz at seizure onset (FA^60−100^) and the significantly activated electrodes (as compared to a baseline) are reported on the patient MRI to provide a 3-D anatomical map of seizure onset and propagation. Statistics can be performed at the group level, between seizures in the same patient or between patients suffering from the same type of epilepsy using normalization of brains to a common anatomical atlas. Such a quantification of FA^60−100^ was proved useful to provide clinicians with objective measurements and localization of the epileptic circuits (7, 20–23). However, whether the EM method reveals FA in any forms of focal epilepsies, whether it gives similar information as the traditional visual approach, and whether it helps to better delineate the EZ remain crucial issues.

To this aim, we conducted a study in a series of 21 consecutive patients suffering from of drug-resistant focal epilepsy and who underwent a SEEG study before surgery. SEEG recordings were analyzed both visually and using the EM approach, and the prognostic significance of removing areas exhibiting FA^60−100^ at seizure onset was evaluated.

## Materials and Methods

This retrospective study was carried out in accordance with the recommendations of Direction de la Recherche Clinique of INSERM with written informed consent from all subjects or their representatives. The protocol was approved by the Comité d'Evaluation Ethique de l'INSERM IRB00003888 (protocol number 14-140).

### Inclusion Criteria

For the purpose of this retrospective study, 21 of 27 consecutive patients suffering from drug-resistant focal seizures and studied by SEEG at Grenoble-Alpes University Hospital as part of their presurgical evaluation were selected according to the following criteria: (i) recording of at least one spontaneous seizure during SEEG investigation; (ii) surgery performed after SEEG investigation; (iii) at least 24 months of post-operative follow-up.

### Patients' Characteristics

All patients were suffering from drug-resistant focal epilepsy, the operability of which could not be decided on the basis of non-invasive procedures only. These latter included in all cases high-resolution MRI, scalp-video-EEG monitoring, and neuropsychological tests. ^18^FDG PET was performed in 15 cases.

There were 12 males and nine females in the series of patients, whose mean age at SEEG investigation was 26.9 years (range 6–45 years). Mean age at seizure onset was 11.3 years (range 0–25 years), and mean duration of epilepsy before SEEG was 15.7 years (range 4–29 years). MRI demonstrated different kind of lesions in 13 patients, of whom four had previously undergone unsuccessful surgery before SEEG procedure (#2, 8, 9, 14). MRI was normal in eight.

### SEEG Investigation

Ten to 18 electrodes were implanted in each patient (mean: 14) in stereotaxic conditions, according to the SEEG methodology developed in our group (24). The targets and number of intracerebral electrodes was tailored in each individual depending on the suspected origin of seizures. Preoperative targeting was performed using 3-D T1 brain MRI computed with a stereotactic software (VoximR, IVS solution, Germany), and using a stereotaxic and stereoscopic digitalized arteriography to determine avascular trajectories of the electrodes. Insertion of the electrodes (DIXI Medical, Besançon, France; diameter of 0.8 mm; 10–18 contacts, 2 mm length, 1.5 mm apart) was guided by a robotic arm (Neuromate, ISS, France) that was connected to the stereotactic frame and driven by the stereotactic software. All patients also underwent a peri-implantation MRI that allowed the direct visualization of the trajectory of each electrode.

Intracerebral recordings were conducted extra-operatively in chronic conditions (1–3 weeks) with reduced medication using an audio-video-EEG monitoring system (Micromed, Treviso, Italy) that allowed to record simultaneously up to 128 contacts, with a sampling rate of 512 Hz, and an acquisition band-pass filter between 0.1 and 200 Hz. Depth EEG activity was displayed between contiguous contacts at different levels along the axis of each electrode, and analysis of SEEG traces was done visually to delineate the epileptogenic region.

### Surgery

Surgery was performed according to the visual analysis of SEEG data. Particular attention was paid to the classical fast discharge recorded prior to the clinical onset of the seizure to delineate the cortical areas to be removed. At the time of the clinical decision, EM analysis was not available.

The extent of the resection was assessed on a 3-D T1 post-operative MRI usually performed 3 months after surgery. Images were normalized into the MNI coordinates to be further compared with EM.

Pathological examination was available in all cases and postoperative outcome was assessed at the last evaluation, according to Engel's classification (25).

## Data Analysis

### Visual Analysis of SEEG Data

All the SEEG-recorded spontaneous seizures were reviewed visually by one SEEG expert (LM) to delineate the EZ. When more than 10 seizures were recorded in one patient, only the first ten were analyzed. The EZ was defined as the cortical area(s) exhibiting clear SEEG changes within the first 4 s of the seizure onset. This choice of 4 s. was made in order to have an information of the initial network organization of the ictal discharge, and to allow comparison between visual and quantitative analysis. SEEG changes were considered as relevant when they occurred prior to the clinical onset of the seizure, and when they consisted in a fast synchronizing discharge (low voltage fast activity, or fast discharge of spikes). All SEEG contacts exhibiting this SEEG pattern were considered as being part of the EZ, and labeled as visually involved (VA+). SEEG contacts which did not exhibit this pattern were considered as not being part of the EZ and were therefore labeled as VA–.

### Quantitative Analysis of SEEG Data

All the SEEG-recorded seizures were independently processed (ASJ) using a quantitative analysis by epileptogenicity mapping, keeping the same definition of the EZ as for VA. The clinician (ASJ) was only aware of the time at seizure onset as defined by VA (done by clinician LM) in order to process the signal during the same time period. Epileptogenicity maps (EM) were obtained according to our previous study (7), using ImaGIN (https://f-tract.eu/index.php/software/imagin/), a homemade toolbox compatible with the Statistical Parametric Mapping (SPM) software (http://www.fil.ion.ucl.ac.uk/spm). The same code has been recently implemented in the Brainstorm software and tutorials for epileptogenicity mapping can be currently found in Brainstorm to facilitate replication studies (https://neuroimage.usc.edu/brainstorm/Tutorials/Epileptogenicity).

Due to the sampling rate of SEEG recordings (512 Hz), we found reasonable to set the superior limit of FA at 100 Hz. The presence of ictal FA^60−100^ was quantified for each electrode contact, by transforming the raw-SEEG signal into a time (*t*)/frequency (*f*) chart of power *P*. This time frequency chart of P(*t, f*) began with seizure onset and was analyzed for the first 4 s (Δ) with a temporal resolution *dt* of 100 ms and a spectral resolution of 1 Hz. A 15–20 s baseline period (Δ_b_) was selected within a 20–60 s window before seizure onset, without artifacts, for each seizure. FA^60−100^ power, *P* averaged in the frequency band 60–100 Hz, was computed for Δ/*dt* time points during the seizure onset, and compared with Δ_*b*_*/dt* samples from the baseline, for each electrode channel. These data were log-transformed to obtain a normalized distribution before the statistical test. Results of the log transform were interpolated on isotropic voxels of 3 mm, in order to represent the data obtained on the patient anatomical MRI normalized in the MNI referential with a good precision. Statistics of the difference of images of log-power of FA^60−100^ between seizure onset and baseline were obtained with a sample *t*-test, family-wise error (FWE) corrected to allow multiple comparisons. Epileptogenicity was defined as the result of this statistical test, i.e., the *t*-value of the differences in smoothed log-power between seizure and baseline. The *p*-value was set at 0.05 (FWE) to define statistical significance in maps of epileptogenicity. SEEG contacts that proved significantly activated, i.e., contained in the volume defined by significant epileptogenicity values, were labeled as EM+, those that did not exhibit significant activation were labeled as EM–. For each patient, 3-D maps of the most significant global maxima of FA^60−100^ at seizure onset were produced and represented on the patient anatomical 3-D T1 MRI, normalized in the MNI referential. The maps were performed for all individual seizures and at the patient level (thereafter defined as seizure group level).

### Comparison Between Quantitative and Visual Analyses

An overlap ratio (OR) was first calculated for each seizure in order to evaluate whether VA and EM gave similar information. OR was defined as the number of SEEG contacts labeled as [VA+EM+] or [VA–EM–], divided by the total number of SEEG contacts (Table 1). Then, two additional ratios of overlap were calculated in order to evaluate the concordance of the two methods for defining the EZ (EZ + R), or for defining the regions that were spared at seizure onset (EZ – R). They were calculated as follows: EZ + R = [VA+EM+]/([VA+EM+] + [VA+EM–] + [VA–EM+]); EZ–R = [VA–EM–]/([VA–EM–] + [VA–EM+] + [VA+EM–]).

**Table 1:** Comparison between visual and quantitative analysis.

**Pts**	**Epileptogenic**	**Szrs**	**Contacts**	**VA+**	**EM+**	**VA+**	**VA–**	**VA+**	**VA–**	**OR**	**EZ+**	**EZ–R**	**Engel's**	**FR**
	**zone**		**number**	**VA+**	**EM+**	**EM+**	**EM–**	**EM–**	**EM+**	**(%)**	**R (%)**	**(%)**	**score**	**(%)**
1^*^	R F (premotor)	1–3	126	8.3	14.7	5.7	108.3	2.7	9	90.4	32.8	90.3	Ia	43.22
		1		10	8	9	109	1	7	93.6	52.9	93.2		
		2		8	9	1	109	7	8	87.3	6.3	87.9		
		3		7	19	7	107	0	12	90.7	36.8	89.9		
2	R T+ (T-insular)	4	126	21	40	14	79	7	26	73.8	29.8	70.5	Ic	2.4
3^*^	L F (opercular)	5	125	37	84	35	39	2	49	59.2	40.7	43.3	III	1.5
4^*^	R T+ (T-insular)	6-8	126	12	22.7	3.7	95	8.3	19	78.3	11.9	77.7	IV	4.7
		6		13	0	0	113	13	0	89.7	0	89.7		
		7		11	3	0	112	11	3	88.9	0.2	88.9		
		8		12	65	11	60	1	54	56.3	16	52.2		
5^*^	L F+ (F-insular)	9-11	126	13.7	9.3	4.3	107.3	9.3	5	88.6	23	88.2	II	10.8
		9		18	9	1	100	17	8	80.1	3.8	80.0		
		10		9	2	0	115	9	2	91.2	0	91.3		
		11		14	17	12	107	2	5	94.4	63.2	93.9		
6	L F (opercular)	12-15	125	25	98.3	25	26.7	0	73.2	41.4	25.5	26.7	IV	17.7
		12		34	109	34	16	0	75	40	31.2	17.6		
		13		28	107	28	18	0	79	36.8	26.2	18.6		
		14		21	85	21	40	0	64	48.8	24.7	38.5		
		15		17	92	17	33	0	75	40	18.5	30.6		
7^*^	L Pcx (O mesial)	16-18	126	6.7	12.7	6	112.7	0.7	6.7	94.2	44.8	93.8	II	29.1
		16		7	8	5	116	2	3	96	50	95.9		
		17		9	18	9	108	0	9	92.9	50	92.3		
		18		4	12	4	114	0	8	93.6	33.3	93.4		
8^*^	R F (opercular)	19-28	123	34.1	19.2	76.8	19.2	28	11.6	68.2	66	32.7	IV	17.9
		19		36	116	7	116	0	80	35	8	59.2		
		20		16	6	101	6	16	5	82.9	82.8	22.2		
		21		43	2	78	2	43	2	63.4	63.4	4.3		
		22		68	19	46	19	58	9	45.5	40.7	22.1		
		23		48	0	75	0	48	0	61	61	0		
		24		49	0	74	0	49	0	60.2	60.2	0		
		25		49	8	68	8	47	6	56.9	56.2	13.1		
		26		11	5	109	5	9	3	90.2	90.1	29.4		
		27		10	15	108	15	0	5	95.9	95.6	75.0		
		28		10	21	102	21	0	11	91	90.3	65.6		
9^*^	R T+ (T-insular)	29	126	29	52	28	73	1	24	78.3	52.8	74.5	III	22.6
10	R T (mesial)	30	125	19	9	9	106	10	0	92	47.4	91.4	Ia	52.0
11^*^	L Pcx (O-T)	31-38	126	32.3	79.4	28.5	42.9	3.4	50.9	56.6	34.4	44.1	Ia	18.3
		31		54	95	51	28	3	44	62.7	52	37.3		
		32		12	99	12	27	0	87	30.9	12.1	23.7		
		33		21	42	11	74	10	31	67.5	21.2	64.3		
		34		87	116	83	6	4	33	70.6	69.2	14.0		
		35		26	66	26	60	0	40	68.3	39.4	60.0		
		36		40	71	27	42	13	44	54.8	32.1	42.4		
		37		8	74	8	52	0	66	47.6	10.8	44.1		
		38		10	72	10	54	0	62	50.8	13.9	46.6		
12	L T (mesial)	39	107	18	69	18	39	0	51	52.3	25	43.3	II	28.5
13	R F (extended)	40-42	119	21	63	20	43	7	49	52.9	26.3	43.4	IV	16.3
		40		21	58	21	37	0	61	48.7	25.6	37.8		
		41		32	57	26	31	6	56	47.9	29.5	33.3		
		42		28	74	13	61	15	30	62.2	22.4	57.5		
14	L Pcx (O-T)	43	125	18	38	10	79	8	28	71.2	21.7	68.7	IV	6.2
15^*^	L F (premotor)	44-51	96	29.1	37.5	18.1	47.6	11	19.2	68.5	37.5	61.2	IV	24.4
		44		24	0	0	72	24	0	75	0	75.0		
		45		18	23	9	64	9	14	76	28.1	73.6		
		46		14	43	14	53	0	29	69.8	32.6	64.6		
		47		35	3	3	61	32	0	66.7	8.6	65.6		
		48		36	39	20	41	16	19	63.5	36.4	53.9		
		49		23	69	23	27	0	46	52.1	33.3	37.0		
		50		26	31	21	60	5	10	84.4	58.3	80.0		
		51		57	91	55	3	2	36	60.4	59	7.3		
16	L T (mesial)	52	125	51	34	29	69	22	5	78.4	51.8	71.9	IV	25.8
17^*^	L F (opercular)	53-56	126	21.5	62	21	66	0.5	41	69	33.6	61.4	Ia	16.7
		53		18	66	18	60	0	48	61.9	27.3	55.6		
		54		22	55	22	71	0	33	73.8	40	68.3		
		55		22	61	20	73	2	41	73.8	31.7	62.9		
		56		24	66	24	60	0	42	66.7	36.4	58.8		
18	L F+ (F-T)	57	117	43	94	41	21	2	53	53	42.7	27.6	Ia	41.6
19	R T (mesial)	58-59	124	8.5	4	0	111.5	8.5	4	89.9	0	89.9	IV	26.8
		58		12	0	0	112	12	0	90.3	0	90.3		
		59		5	8	0	111	5	8	89.5	0	89.5		
20	L F (premotor)	60	125	16	115	16	10	0	99	20.8	13.9	9.2	Id	24.4
21	L F (extended)	61-68	126	46.3	52.4	27.2	54.4	19.1	25.1	64.8	38.1	55.2	IV	13.5
		61		41	112	41	13	0	71	42.9	36.6	15.5		
		62		20	28	12	90	8	16	80.9	33.3	78.9		
		63		60	18	6	54	54	12	47.6	8.3	45.0		
		64		58	103	58	23	0	45	64.3	56.3	33.8		
		65		48	14	7	71	41	7	61.9	12.7	59.7		
		66		59	48	36	55	23	12	72.2	50.7	61.1		
		67		23	15	2	90	21	13	73	5.6	72.6		
		68		62	81	56	39	6	25	75.4	64.4	55.7		

Each white row represents one seizure, gray rows represent a group of seizures for one patient. VA, visual analysis (+/–, number (or mean number) of contacts involved/not involved at seizure onset); EM, epileptogenicity map (+/–, number (or mean number) of contacts showing a significant activation/no activation at seizure onset); OR, overlap ratio between VA and EM; EZ+R, overlap ratio evaluating the concordance of the two methods for defining the EZ; EZ-R, overlap ratio evaluating the concordance of the two methods for defining the regions that were spared at seizure onset; FR, FASTectomy ratio;

* *Patients with a positive MRI; F/F+, frontal/frontal+; T/T+, temporal/temporal+; Pcx, posterior cortex; O, occipital*.

We further assessed whether the concordance between VA and EM depended on the type and location of epilepsy, or was associated with the surgical outcome. To do so, and in order to avoid biasing our quantitative analysis (the number of seizures per patient ranged from 1 to 10), we calculated a mean OR per patient, defined as the mean number of SEEG contacts that were both labeled as [VA+ EM+] or [VA– EM–] for the group of seizures, divided by the mean number of SEEG contacts. Similarly, a mean EZ + R and a mean EZ – R were calculated.

Statistical analyses were performed with GraphPad Prism 7.0. We used the Kruskal-Wallis test to compare the different mean ratios and the location of the EZ (frontal/frontal + vs. temporal/temporal+ vs. posterior), and the Mann-Whitney test to compare the different ratios with the type of epilepsy (MRI positive vs. MR negative) and the surgical outcome (class I vs. class II-III-IV).

### Comparison Between Quantitative Analysis and Extent of the Resection

**Figure 1:**
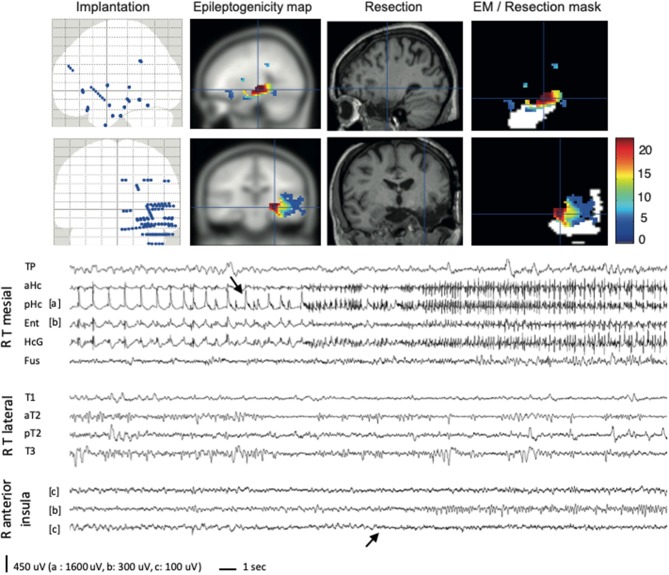
EM method applied to a temporal epilepsy case (#4). For this patient, the semiology pointed to a right temporo-insular involvement, in association with a hippocampal sclerosis. Right temporal and perisylvian implantation was performed. The only seizure recorded started in the hippocampus (first arrow), with an early but discrete visual involvement of the insular cortex (second arrow). EM retrospectively confirmed the hippocampal involvement, but also enhanced the early insular involvement during the seizure. The resection (planned on visual analysis) was limited to the mesial temporal cortex, and FASTectomy ratio was very low (4%). Immediately after surgery, the patient started to have seizures pointing to the right insula (left tingling, throat constriction). The colorbar indicates the epileptogenicity values. Resection mask is in white. R, right; T, temporal; TP, temporal pole; aHc/pHc, anterior/posterior hippocampus; Ent, entorhinal cortex; HcG, parahippocampal gyrus; Fus, fusiform gyrus; T1/T2/T3, first/second/third temporal gyrus.

**Figure 2:**
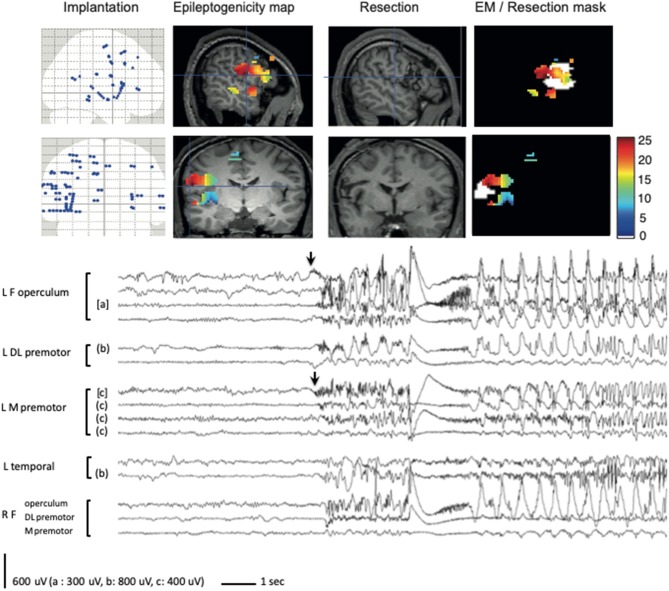
EM method applied to a frontal epilepsy case (#6). A bilateral premotor and opercular implantation was performed, predominating on the left. The fourth seizure recorded is shown, demonstrating a widely extended mesio-lateral network at seizure onset, involving predominantly the left frontal operculum but also the left mesial premotor cortex (arrows). EM applied to the same seizure demonstrated similar significant activation of FA, but enhanced the major involvement (in red) of the frontal operculum. Resection was mainly targeted over the frontal operculum but proved incomplete because of a weak participation of the patient during awake surgery, which did not allow to correctly evaluate language areas. FASTectomy ratio was low (17.7%) and patient was not improved after surgery. The colorbar indicates the epileptogenicity values. Resection mask is in white. R, right; L, left; F, frontal; DL, dorsolateral; M, mesial.

To evaluate the prognostic value of removing the brain regions displaying significant FA^60−100^ at seizure onset, a resection mask was delineated on post-operative 3-D T1 MRI of each patient, co-registered with both anatomical and peri-implantation MRI, allowing to compare the extent of the resection with EM (Figures 1, 2). We then calculated a FASTectomy ratio for each patient, by dividing the sum of the epileptogenicity values of all the voxels with significant FA^60−100^ belonging to the resection mask by the sum of the epileptogenicity values of all voxels of the image with significant FA^60−100^. A ratio of 1 meant that all cortical regions generating FA^60−100^ were resected. The patients were ranked into six groups according to their post-surgical outcome (Engel's class IA to IV). A non-parametric Kruskal-Wallis test was performed to look for any differences between the groups. Significance was set at *p* < 0.05.

## Results

### Global Results

A total number of 68 seizures were analyzed in the 21 patients (mean: 3.2 per patient), with an average number of recording contacts per patient of 122.5 (SD 7.6).

Based on SEEG findings, the EZ proved to be left sided in 13 patients, and right-sided in eight. It was unilobar in 14 cases and multilobar in seven. More precisely, the EZ was frontal (premotor: 3; opercular: 4; extended: 2) or frontal+ (fronto-temporal: 1; fronto-insular: 1) in 11 patients, temporal (mesio-temporal: 4) or temporal+ (temporo-insular: 3) in 7 patients, and posterior in 3 patients (occipital: 1, occipito-temporal: 2).

Surgery was performed in one step in 17 cases, and in two-steps in four cases (#11, #13, #18, #21). In four patients, the EZ could not be fully removed because of functional anatomical constraints (#3, #8, #16) or weak participation of the patient during awake surgery (#6).

Pathological examination found different kind of lesions in 10 of the 13 patients whose MRI proved positive, including focal cortical dysplasia (FCD) in seven, hippocampal sclerosis (HS) in one, nodular heterotopia (NH) in one, and cavernoma in one. Three patients had nonspecific gliosis. In the eight patients without any abnormality on MRI, histological exam revealed a nonspecific gliosis in five, FCD in two, and HS in one.

After a mean post-operative follow-up of 50.1 months (range 24–80 months), seven patients (33%) were in class I (IA: 5, IC: 1, ID: 1), five (24%) were improved (II: 3, III: 2) and nine (43%) were unchanged.

### Epileptogenicity Maps

EM revealed a significant activation in the 60–100 Hz band at seizure onset in all but 5 of the 68 seizures (92.6%), and in at least one seizure in all the 21 patients (Table 1). The five seizures in which EM failed to exhibit any significant changes occurred in 3 of the 13 MRI positive cases, and in one of the eight MRI negative cases. They were recorded in one mesio-temporal case, one temporal+ case, and in two frontal lobe cases.

### Epileptogenicity Maps vs. Visual Analysis

At the seizure level (*n* = 68, Table 1), the mean number of contacts involved at seizure onset was higher using EM (46.0, SD: 37.2) than using VA (27.6, SD: 18.2), and the difference was statistically significant (*p* < 0.0001). OR ranged from 20.8 to 96.0%, with an average of 67.8% (SD: 18.4). These values, however, were biased by the high number of [VA–EM–] contacts (mean: 64.3, SD: 33.5), as compared with the number of [VA+EM+] contacts (mean: 20.7, SD: 17.0). It thus appeared that the concordance of the two methods was better for defining the regions that were spared at seizure onset (EZ–R, mean: 54.6%, SD: 27.6) than for defining the EZ (EZ + R, mean: 34.9%, SD: 23.7), the difference being statistically significant (*p* < 0.001).

**Figure 3:**
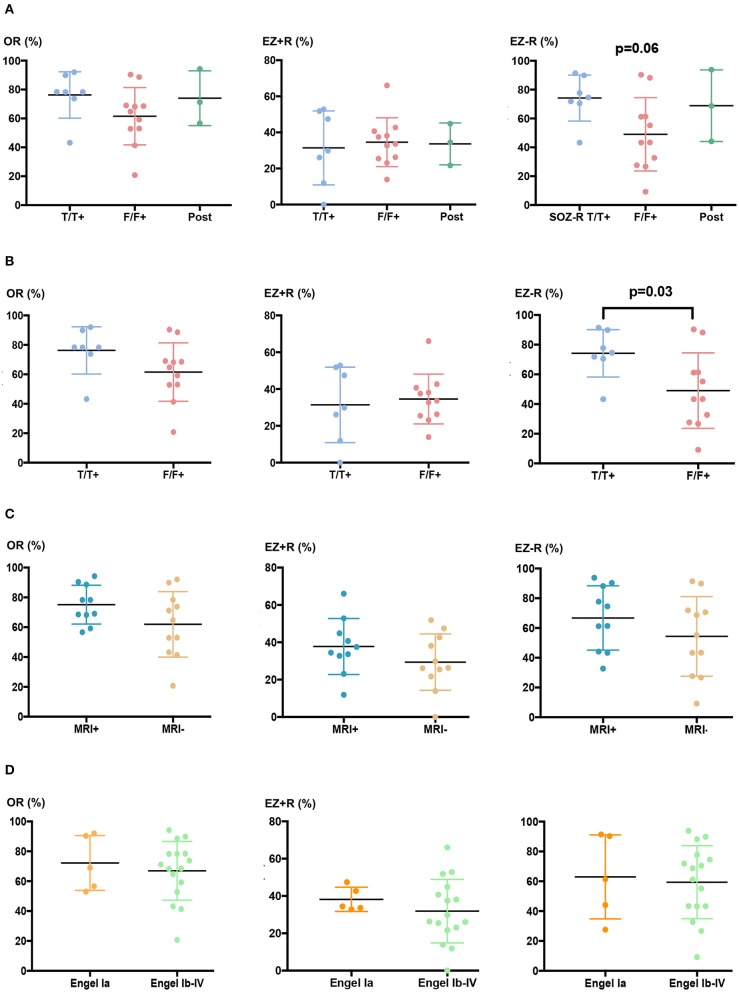
Overlap ratios between visual and quantitative analysis depending on the epilepsy type and surgical outcome. **(A)** Global overlap ratio between the two methods (OR, left) and their concordance to define the EZ (EZ + R, middle) and the regions that were spared at seizure onset (EZ – R, right) for all epilepsy locations (F/F+, frontal/frontal+, T/T+, temporal/temporal+, Pcx, posterior cortex). Mean values of OR, EZ + R, and EZ – R were, respectively 78.3, 33.6, and 74.5% for T/T+ patients (*n* = 7), 64.8, 29.8, and 43.4% for F/F+ patients (*n* = 11), and 71.2, 34.4, and 68.7% for Pcx patients (*n* = 3). **(B)** OR, EZ + R, and EZ–R for F/F+ and T/T+ patients only. **(C)** OR, EZ + R, and EZ – R in MRI+ (*n* = 13) or MRI- (*n* = 8) patients. Mean values were, respectively 73.6, 35.9, and 67.9 for MRI+ patients, and 64.8, 26.3, and 65.2% for MRI- patients. **(D)** OR, EZ + R, and EZ – R in seizure-free (class Ia, *n* = 5) and not seizure-free (class Ic to IV *n* = 16) patients. Mean values of OR, EZ + R, and EZ – R were, respectively 69.0, 34.4, and 61.4% for patients in class Ia, and 69.8, 28.1, and 64.9% for patients in class Ib to IV.

Similar findings were found at the patient level (*n* = 21, Table 1). The concordance between visual and quantitative analysis for defining the regions spared at seizure onset was better in temporal/temporal+ (mean: 74.5%) than in the other forms of epilepsy, a difference that almost reached significance (*p* = 0.06) (Figure 3A). This tendency was further confirmed when comparing only temporal/temporal+ patients with frontal/frontal+ patients (*p* = 0.036) (Figure 3B). The presence or absence of MRI abnormalities showed no difference whatever the ratio studied (Figure 3C).

### Epileptogenicity Maps and Surgical Outcome

#### Relationships Between Overlap Ratios and Surgical Outcome

To evaluate whether the concordance between quantitative and visual analysis might have a prognostic significance, OR, EZ + R, and EZ–R were compared between patients who were completely seizure-free after surgery (class Ia) and those who were not (class Ib to IV). No significant difference was found between the two groups (Figure 3D), even after excluding the four patients in whom resective surgery was incomplete because of functional anatomical constraints.

#### Correlation Between FASTectomy Ratio and Seizure Outcome

**Figure 4:**
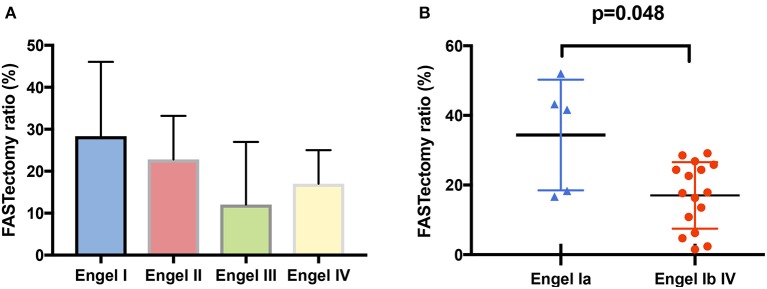
FASTectomy ratios depending on post-operative outcome. **(A)** FASTectomy ratio was globally higher in class I patients (mean: 28.4%), as compared with class II (mean: 22.8%), class III (mean: 12.1%), and class IV (men: 17%) patients. **(B)** The mean FASTectomy ratio was higher in completely seizure-free patients (class Ia) than in non seizure-free patients (class Ib to IV) and the difference was statistically significant (*p* = 0.048, Mann-Whitney test). Nine non seizure-free patients, however, had a higher FASTectomy ratio than some of class Ia patients.

The mean FASTectomy ratio was higher in Engel I patients (mean = 28.4%), than in Engel II (mean 22.8%), Engel III (mean 12.1%), and Engel IV (mean 17%) patients (Figure 4A). Statistical comparison was performed between patients who were completely seizure-free (Engel Ia) and those who were not (Engel Ib to IV), and the difference was statistically significant (*p* = 0.048) (Figure 4B). This suggests that quantitative analysis using EM might be helpful to tailor the resection. The small number of patients does not allow us to evaluate whether the FASTectomy ratio correlated with the surgical outcome depending on the epilepsy type.

## Discussion

The last decade, a growing field of research has focused on intracranially-recorded *interictal events*, named high-frequency oscillations (HFO, 80–500 Hz), that could be a relevant signature of the EZ (26). Studying these events, although particularly interesting, was not the purpose of the present work. We rather concentrated here on another research field focused on *ictal events*, that we called fast activities (FA) to avoid any confusion and because their oscillatory character has not been clearly established.

The occurrence of FA is a common finding at seizure onset in patients submitted to iEEG, especially in seizures of neocortical origin (4). Different pathological substrates may share this pattern (2, 3), that might be associated with a more favorable surgical outcome (5, 11, 15–18). Different computer-based signal analysis methods have thus been developed for objectively quantifying ictal FA in different frequency ranges, based on automatic (9), semi-automatic (19) or visual (5, 7, 10, 11, 15, 18) detection of the seizure onset. These approaches may also incorporate other variables such as amplitude, mobility, and complexity (5), attenuation of slower alpha-theta activities and/or time course of FA (18, 19), slow polarizing shift and voltage depression (9) or spikes preceding multiband FA concurrent with suppression of low frequencies (10). Some of these methods, recently compared in a very small series of patients, have been proved to converge for detecting FA when traditional visual inspection was successful, whereas they showed discordant results and did not provide relevant information in comparison to traditional analyses in patients with more complex EZ (27).

In this context, our study aimed at assessing in more details the EM method (7) with the 3-fold purpose to evaluate (i) whether EM could detect ictal FA in different forms of focal epilepsies, (ii) whether EM provided similar information than visual analysis and (iii) whether EM might help for the surgical decision.

### Can ictal FA Be Quantitatively Detected in All Epilepsy Types?

Our study, although conducted in a small series of patients (*n* = 21), takes advantage of the cohort heterogeneity both for epilepsy location and pathological substrates. A significant ictal FA^60−100^ activation was found at least in one seizure for all patients, and in 92.6% of all seizures, although age at SEEG, epilepsy duration, underlying pathology and seizure localization widely varied across patients (Table 1). EM thus appears as a highly sensitive method to detect FA in the 60–100 Hz range at seizure onset whatever the epilepsy type, which encourages the use of computerized approaches focused on this frequency band.

In 5 of the 68 seizures, however, recorded in four patients, we failed to identify any significant FA^60−100^ activation, even though FA were visually identified. This limitation, already reported with other methods (14, 28–30), can be partly solved by performing statistics at the seizure group level in the same patient, which requires the recording of multiple seizures in each individual. Nevertheless, the question of why EM miss some ictal FA remains opened, and there could be different reasons for this finding: (i) the 4 s sliding window that we used was too short to detect significant FA; Wu et al. for instance, found that FA started 5–55 s after the SEEG onset in most (70%) of mesio-temporal lobe seizures (30); these authors, however, used a visual definition of the seizure onset very different from ours, since they looked at rhythmic sinusoidal activity or repetitive spikes rather than at fast rhythms; (ii) the frequency range that we considered was too high to detect all FA that were visually identified; indeed, it has been shown that focal seizures—mainly of mesio-temporal lobe origin—often start with FA in the beta and low gamma bands (5, 17, 31); nevertheless, only one of the five seizures where EM failed to detect FA^60−100^ started in MTL structures, and EM was positive in four of the five MTL seizures recorded; (iii) EM detected only statistically significant FA^60−100^ activation with respect to a baseline chosen very close (20–60 s) to the ictal onset, so that a subtle increase of FA could have been underestimated; in particular, interictal HFO that may occur or increase near the time of seizure onset (14, 32, 33) could produce an interference with the EM method, although their frequency range is usually above 80 Hz; similarly, the filtering of interictal spikes may produce FA during the baseline period (34) and therefore could falsely attenuate the ictal increase of FA^60−100^. Choosing a baseline in the postictal phase could overcome this problem, especially in FCD cases where the structures involved at seizure onset usually display a strong postictal depression (35).

### Are Computers Better Than the Human Eye to Detect FA at Seizure Onset?

To the best of our knowledge, none of the signal analysis methods has provided results that strictly matched the traditional visual identification of the EZ. Our study, focused on FA only, does not escape this assumption. The mean overlap ratio (OR) between visual (VA) and quantified (EM) analyses, as assessed blindly, was 67.8% (20.8–96.0%) for the 68 seizures, and 68.6% (20.8–94.2%) for the 21 patients. As expected, no difference was found between MRI+ and MRI– patients. This concordance was close to the one reported by Gnatkovsky et al. (9), whose computer-assisted method—that included both FA, flattening and slow polarizing shift—matched with the visually-defined EZ in 74% of the 14 studied patients. It is also closed to the study of Wu et al. (30) which found, in 61 mesio-temporal lobe seizures, a 70% spatial correlation between the EZ and ictal FA as assessed only visually. Discordant results, however, accounted for a substantial number of recording sites and this might be due, as stated above, to the difficulty to detect FA in the high gamma range using the traditional visual inspection, and/or to possible high fractions of FA that might occur during the baseline period in the region from where seizures start. This can be due also that VA was assessed by one clinician only, which makes impossible any interrater reliability that is known to be moderate for VA of iEEG signals [see for instance (36) for seizure identification during iEEG].

Nevertheless, our work interestingly showed that the concordance between VA and EM was better for defining the regions spared at seizure onset than those from which seizure arose (*p* < 0.001), especially in temporal/temporal+ patients. This implies that EM might have a good negative predictive value, which could be helpful for minimizing the resection. In the contrary, the two methods were much more discordant to define the EZ, with a mean number of electrode contacts involved at seizure onset significantly higher with EM than with VA (*p* = 0.04). This large EM activation, that might suggest a limited positive predictive value of EM, could be due to the normalization step and spatial smoothing required for the correction of multiple comparisons that we used with our method. It could be due also, as already mentioned, to SEEG features that remain sub-threshold to VA and can be revealed only by quantitative measurements.

### Can Surgical Outcome Be Improved by Using Quantitative Analysis of ictal FA?

The most common criterion for determining whether a particular ictal onset pattern is helpful in epilepsy surgery is to evaluate its impact on surgical outcome. A recent meta-analysis found that the low voltage FA pattern was associated with good outcome in various forms of epilepsies, especially when neocortical and focal (4). A Cochrane review, however, identified only two studies (11 patients in total) which used ictal FA in making decisions about epilepsy surgery, with no reliable conclusions due to methodological limitations and the small sample size (37). There is therefore only sparse evidence on the value of ictal FA alone to guide surgical resection, in as much as their most relevant frequency range is not known, and—more importantly—that they do not consider the whole seizure pattern, as recently emphasized (10).

Our study was conducted in a cohort of particularly complex cases, of whom 8/21 (38%) had a negative MRI, 14/21 had an extra-temporal EZ, 7/21 (33%) had a multilobar EZ, and 4/21 (26.1%) had previously undergone unsuccessful surgery. As expected, surgical outcome was quite poor (only 38% of patients had a class I outcome), and the aim of our study was precisely to define whether the use of FA quantification could have added different information. We showed first that although the spatial distribution of the visually-defined EZ and ictal FA^60−100^ did not fully overlap, such a discrepancy did not have any prognostic significance for seizure outcome. This applied both for the detection of regions involved at seizure onset and for the identification of regions that were spared. Our results suggest that computer-based signal analysis methods cannot substitute for visual analysis, but might complement the traditional visual inspection in a way that will need further clarification. A second important result of our study was to show that the FASTectomy ratio significantly correlated with seizure outcome, which therefore underlines that evaluation of FA^60−100^ at seizure onset might help to adapt surgery. Interestingly, none of the four patients of whom some of the seizures did not exhibit any FA at EM analysis were seizure-free after surgery, which could represent a “red flag” indicating that the EZ was incompletely sampled. This result, however, does not mean that ictal FA is the only electrophysiological factor of prognostic significance, as illustrated by a recent SEEG study showing that although FA at seizure onset was associated with favorable outcome in patients with FCD and neurodevelopmental tumors, the completeness of the EZ resection was the sole independent predictive variable (16). Our study does not mean either that ictal FA can delineate alone the brain areas to be resected. More complex ictal time-frequency patterns, including interictal to ictal transition, FA activations and low frequency suppressions, and seizure evolution, could be more reliable to delineate the EZ, as recently shown by Grynenko et al. (10). Still, other SEEG data such as early seizure spread, interictal activity, post-ictal depression and electrical stimulation results are also considered for the surgical decision (38). Also, a growing amount of data suggests that other relevant biomarkers of the EZ do exist, namely interictal HFO, as showed by a recent meta-analysis that found a higher resection ratio for HFO in seizure-free vs. non seizure-free patients (39). Importantly, existing data, including ours, cannot answer to the crucial clinical question of whether a patient will become seizure-free if the FASTectomy or HFOectomy ratios are high. Our results, indeed, although significant at the group level, showed that some of the non seizure-free patients exhibited a FASTectomy ratio higher than in the seizure-free group (Table 1), a finding also reported when studying interictal HFO (40).

## Data Availability Statement

The pre-processed data supporting the conclusions of this manuscript will be made available by the authors, without undue reservation, to any qualified researcher.

## Ethics Statement

This retrospective study was carried out in accordance with the recommendations of Direction de la Recherche Clinique of INSERM with written informed consent from all subjects. The protocol was approved by the Comité d'Evaluation Ethique de l'INSERM IRB00003888 (protocol number 14-140).

## Author Contributions

A-SJ: collected data, performed data analysis, and wrote the manuscript. OD: designed the study, performed data analysis, and wrote the manuscript. LM: collected data and performed data analysis. FB: wrote the manuscript. SC: collected data. PK: designed the study, collected data, performed data analysis, and wrote the manuscript.

### Conflict of Interest

The authors declare that the research was conducted in the absence of any commercial or financial relationships that could be construed as a potential conflict of interest.

## References

[1] Bulacio JC Jehi L Wong C Gonzalez-Martinez J Kotagal P Nair D. Long-term seizure outcome after resective surgery in patients evaluated with intracranial electrodes. Epilepsia. (2012) 53:1722–30. 10.1111/j.1528-1167.2012.03633.x 22905787

[2] Lagarde S Buzori S Trébuchon A Carron R Scavarda D Milh M. The repertoire of seizure onset patterns in human focal epilepsies: determinants and prognostic values. Epilepsia. (2019) 60:85–95. 10.1111/epi.14604 30426477

[3] Perucca P Dubeau F Gotman J. Intracranial electroencephalographic seizure-onset patterns: effect of underlying pathology. Brain. (2014) 137(Pt 1):183–96. 10.1093/brain/awt299 24176980

[4] Singh S Sandy S Wiebe S. Ictal onset on intracranial EEG: do we know it when we see it? State of the evidence. Epilepsia. (2015) 56:1629–38. 10.1111/epi.13120 26293970

[5] Alarcón G Binnie CD Elwes RD Polkey CE. Power spectrum and intracranial EEG patterns at seizure onset in partial epilepsy. Electroencephalogr Clin Neurophysiol. (1995) 94:326–37. 10.1016/0013-4694(94)00286-T 7774519

[6] Allen PJ Fish DR Smith SJ. Very high-frequency rhythmic activity during SEEG suppression in frontal lobe epilepsy. Electroencephalogr Clin Neurophysiol. (1992) 82:155–9. 10.1016/0013-4694(92)90160-J 1370786

[7] David O Blauwblomme T Job A-S Chabardès S Hoffmann D Minotti L. Imaging the seizure onset zone with stereo-electroencephalography. Brain. (2011) 134(Pt 10):2898–911. 10.1093/brain/awr238 21975587

[8] Fisher RS Webber WR Lesser RP Arroyo S Uematsu S. High-frequency EEG activity at the start of seizures. J Clin Neurophysiol. (1992) 9:441–8. 10.1097/00004691-199207010-00012 1517412

[9] Gnatkovsky V de Curtis M Pastori C Cardinale F Russo Lo G Mai R. Biomarkers of epileptogenic zone defined by quantified stereo-EEG analysis. Epilepsia. (2014) 55:296–305. 10.1111/epi.12507 24417731

[10] Grinenko O Li J Mosher JC Wang IZ Bulacio JC Gonzalez-Martinez J. A fingerprint of the epileptogenic zone in human epilepsies. Brain. (2018) 141:117–31. 10.1093/brain/awx306 29253102 PMC5837527

[11] Nariai H Nagasawa T Juhász C Sood S Chugani HT Asano E. Statistical mapping of ictal high-frequency oscillations in epileptic spasms. Epilepsia. (2011) 52:63–74. 10.1111/j.1528-1167.2010.02786.x 21087245 PMC3051422

[12] Wendling F Bartolomei F Bellanger JJ Bourien J Chauvel P. Epileptic fast intracerebral EEG activity: evidence for spatial decorrelation at seizure onset. Brain. (2003) 126(Pt 6):1449–59. 10.1093/brain/awg144 12764064 PMC2040489

[13] Worrell GA Parish L Cranstoun SD Jonas R Baltuch G Litt B. High-frequency oscillations and seizure generation in neocortical epilepsy. Brain. (2004) 127(Pt 7):1496–506. 10.1093/brain/awh149 15155522

[14] Jirsch JD Urrestarazu E LeVan P Olivier A Dubeau F Gotman J. High-frequency oscillations during human focal seizures. Brain. (2006) 129(Pt 6):1593–608. 10.1093/brain/awl085 16632553

[15] Usui N Terada K Baba K Matsuda K Usui K Tottori T. Significance of very-high-frequency oscillations (over 1,000Hz) in epilepsy. Ann Neurol. (2015) 78:295–302. 10.1002/ana.24440 25974128

[16] Lagarde S Bonini F McGonigal A Chauvel P Gavaret M Scavarda D. Seizure-onset patterns in focal cortical dysplasia and neurodevelopmental tumors: relationship with surgical prognosis and neuropathologic subtypes. Epilepsia. (2016) 57:1426–35. 10.1111/epi.13464 27406939

[17] Lee SA Spencer DD Spencer SS. Intracranial EEG seizure-onset patterns in neocortical epilepsy. Epilepsia. (2000) 41:297–307. 10.1111/j.1528-1157.2000.tb00159.x 10714401

[18] Ochi A Otsubo H Donner EJ Elliott I Iwata R Funaki T. Dynamic changes of ictal high-frequency oscillations in neocortical epilepsy: using multiple band frequency analysis. Epilepsia. (2007) 48:286–96. 10.1111/j.1528-1167.2007.00923.x 17295622

[19] Bartolomei F Chauvel P Wendling F. Epileptogenicity of brain structures in human temporal lobe epilepsy: a quantified study from intracerebral EEG. Brain. (2008) 131(Pt 7):1818–30. 10.1093/brain/awn111 18556663

[20] Blauwblomme T Kahane P Minotti L Grouiller F Krainik A Vercueil L. Multimodal imaging reveals the role of γ activity in eating-reflex seizures. J Neurol Neurosurg Psychiatr. (2011) 82:1171–3. 10.1136/jnnp.2010.212696 21097547 PMC3338065

[21] Blauwblomme T David O Minotti L Job A-S Chassagnon S Hoffman D. Prognostic value of insular lobe involvement in temporal lobe epilepsy: a stereoelectroencephalographic study. Epilepsia. (2013) 54:1658–67. 10.1111/epi.12260 23848549

[22] Job A-S De Palma L Principe A Hoffmann D Minotti L Chabardès S. The pivotal role of the supplementary motor area in startle epilepsy as demonstrated by SEEG epileptogenicity maps. Epilepsia. (2014) 55:e85–8. 10.1111/epi.12659 24902865

[23] Lamarche F Job A-S Deman P Bhattacharjee M Hoffmann D Gallazzini-Crépin C. Correlation of FDG-PET hypometabolism and SEEG epileptogenicity mapping in patients with drug-resistant focal epilepsy. Epilepsia. (2016) 57:2045–55. 10.1111/epi.13592 27861778 PMC5214566

[24] Kahane P Minotti L Hoffmann D Lachaux J-P Ryvlin P Rosenow F Invasive EEG in the definition of the seizure onset zone: depth electrodes. In: Rosenow F Lüders HO editors. Handbook of Clinical Neurophysiology. Amsterdam: Elsevier BV (2004). p. 109–33.

[25] Engel JJ Van Ness PC Rasmussen TB Ojemann LM Outcome with respect to epileptic seizures. In: Engel JJ editor. Surgical Treatment of the Epilepsies. 2nd ed New York, NY: Raven Press (1993). p. 609–21.

[26] Frauscher B Bartolomei F Kobayashi K Cimbalnik J van t Klooster MA Rampp S. High-frequency oscillations: the state of clinical research. Epilepsia. (2017) 58:1316–29. 10.1111/epi.13829 28666056 PMC5806699

[27] Andrzejak RG David O Gnatkovsky V Wendling F Bartolomei F Francione S. Localization of epileptogenic zone on pre-surgical intracranial EEG recordings: toward a validation of quantitative signal analysis approaches. Brain Topogr. (2014) 28:832–7. 10.1007/s10548-014-0380-8 24929558

[28] Fujiwara H Greiner HM Lee KH Holland-Bouley KD Seo JH Arthur T. Resection of ictal high-frequency oscillations leads to favorable surgical outcome in pediatric epilepsy. Epilepsia. (2012) 53:1607–17. 10.1111/j.1528-1167.2012.03629.x 22905734 PMC3520059

[29] Park S-C Lee SK Che H Chung CK. Ictal high-gamma oscillation (60-99 Hz) in intracranial electroencephalography and postoperative seizure outcome in neocortical epilepsy. Clin Neurophysiol. (2012) 123:1100–10. 10.1016/j.clinph.2012.01.008 22391040

[30] Wu S Kunhi Veedu HP Lhatoo SD Koubeissi MZ Miller JP Lüders HO. Role of ictal baseline shifts and ictal high-frequency oscillations in stereo-electroencephalography analysis of mesial temporal lobe seizures. Epilepsia. (2014) 55:690–8. 10.1111/epi.12608 24725106

[31] Bartolomei F Wendling F Regis J Gavaret M Guye M Chauvel P. Pre-ictal synchronicity in limbic networks of mesial temporal lobe epilepsy. Epilepsy Res. (2004) 61:89–104. 10.1016/j.eplepsyres.2004.06.006 15451011

[32] Khosravani H Mehrotra N Rigby M Hader WJ Pinnegar CR Pillay N. Spatial localization and time-dependant changes of electrographic high frequency oscillations in human temporal lobe epilepsy. Epilepsia. (2009) 50:605–16. 10.1111/j.1528-1167.2008.01761.x 18717704

[33] Zijlmans M Jacobs J Zelmann R Dubeau F Gotman J. High frequency oscillations and seizure frequency in patients with focal epilepsy. Epilepsy Res. (2009) 85:287–92. 10.1016/j.eplepsyres.2009.03.026 19403269 PMC3769287

[34] Bénar CG Chauvière L Bartolomei F Wendling F. Pitfalls of high-pass filtering for detecting epileptic oscillations: a technical note on “false” ripples. Clin Neurophysiol. (2010) 121:301–10. 10.1016/j.clinph.2009.10.019 19955019

[35] Chassoux F Devaux B Landre E Turak B Nataf F Varlet P. Stereoelectroencephalography in focal cortical dysplasia: a 3D approach to delineating the dysplastic cortex. Brain. (2000) 123(Pt 8):1733–51. 10.1093/brain/123.8.1733 10908202

[36] Quigg M Sun F Fountain NB Jobst BC Wong VSS Mirro E. Interrater reliability in interpretation of electrocorticographic seizure detections of the responsive neurostimulator. Epilepsia. (2015) 56:968–71. 10.1111/epi.12998 25895054 PMC5008166

[37] Gloss D Nolan SJ Staba R The role of high-frequency oscillations in epilepsy surgery planning. Cochrane Database Syst Rev. (2014) 52:CD010235 10.1002/14651858.CD010235.pub2 PMC4248664 24431136

[38] Kahane P Landré E Minotti L Francione S Ryvlin P. The Bancaud and Talairach view on the epileptogenic zone: a working hypothesis. Epileptic Disord. (2006) 8(Suppl. 2):S16–26. 17012069

[39] Höller Y Kutil R Klaffenböck L Thomschewski A Höller PM Bathke AC. High-frequency oscillations in epilepsy and surgical outcome. A meta-analysis. Front Hum Neurosci. (2015) 9:574. 10.3389/fnhum.2015.00574 26539097 PMC4611152

[40] Dümpelmann M Jacobs J Schulze-Bonhage A. Temporal and spatial characteristics of high frequency oscillations as a new biomarker in epilepsy. Epilepsia. (2015) 56:197–206. 10.1111/epi.12844 25556401

